# Effectiveness of Internet-Based Cognitive Behavior Therapy (Fatigue in Teenagers on the Internet) for Adolescents With Chronic Fatigue Syndrome in Routine Clinical Care: Observational Study

**DOI:** 10.2196/24839

**Published:** 2021-08-13

**Authors:** Eline Albers, Linde N Nijhof, Emma E Berkelbach van der Sprenkel, Elise M van de Putte, Sanne L Nijhof, Hans Knoop

**Affiliations:** 1 Department of Pediatrics Wilhelmina Children's Hospital University Medical Center Utrecht, Utrecht University Utrecht Netherlands; 2 Department of Medical Psychology Amsterdam University Medical Centers, University of Amsterdam Amsterdam Public Health Research Institute Amsterdam Netherlands

**Keywords:** Fatigue in Teenagers on the Internet, cognitive behavior therapy, fatigue, chronic fatigue syndrome, adolescents, implementation

## Abstract

**Background:**

Internet-based cognitive behavior therapy (I-CBT) for adolescents with chronic fatigue syndrome/myalgic encephalomyelitis (CFS/ME) has been shown to be effective in a randomized controlled trial (RCT; Fatigue in Teenagers on the Internet [FITNET]). FITNET can cause a significant reduction in fatigue and disability.

**Objective:**

We aimed to investigate whether FITNET treatment implemented in routine clinical care (IMP-FITNET) was as effective, using the outcomes of the FITNET RCT as the benchmark.

**Methods:**

Outcomes of CFS/ME adolescents who started IMP-FITNET between October 2012 and March 2018 as part of routine clinical care were compared to the outcomes in the FITNET RCT. The primary outcome was fatigue severity assessed posttreatment. The secondary outcomes were self-reported physical functioning, school attendance, and recovery rates. Clinically relevant deterioration was assessed posttreatment, and for this outcome, a face-to-face CBT trial was used as the benchmark. The attitude of therapists toward the usability of IMP-FITNET was assessed through semistructured interviews. The number of face-to-face consultations during IMP-FITNET was registered.

**Results:**

Of the 384 referred adolescents with CFS/ME, 244 (63.5%) started IMP-FITNET, 84 (21.9%) started face-to-face CBT, and 56 (14.6%) were not eligible for CBT. Posttreatment scores for fatigue severity (mean 26.0, SD 13.8), physical functioning (mean 88.2, SD 15.0), and full school attendance (mean 84.3, SD 26.5) fell within the 95% CIs of the FITNET RCT. Deterioration of fatigue and physical functioning after IMP-FITNET was observed at rates of 1.2% (n=3) and 4.1% (n=10), respectively, which is comparable to a waiting list condition (fatigue: 1.2% vs 5.7%, χ^2^_1_=3.5, *P*=.06; physical functioning: 4.1% vs 11.4%, χ^2^_1_=3.3, *P*=.07). Moreover, 41 (16.8%) IMP-FITNET patients made use of face-to-face consultations.

**Conclusions:**

IMP-FITNET is an effective and safe treatment for adolescents with CFS/ME in routine clinical care.

## Introduction

Chronic fatigue syndrome/myalgic encephalomyelitis (CFS/ME) is a disabling condition in which patients have severe, medically unexplained, and persistent (>6 months) fatigue, resulting in impairment of functioning [1]. The prevalence of CFS/ME in adolescents is 0.11% to 1.29% in Dutch and British adolescent populations, with a female-to-male ratio of 2:1 to 5:1 [2,3]. In adolescents, CFS/ME often has a chronic course, leading to school absence, and has long-term detrimental effects on social and academic development [4,5].

The etiology of CFS/ME is unknown. The context of the biopsychosocial model defines individual predisposing, precipitating, and perpetuating factors that provoke and maintain severe fatigue and disability [6]. Face-to-face cognitive behavior therapy (CBT) aimed at fatigue-maintaining factors has been tested in several randomized controlled trials (RCTs) and leads to a significant reduction in fatigue and an improvement in physical functioning and school attendance [4,7,8]. An internet-based format of CBT for adolescents with CFS/ME, named Fatigue in Teenagers on the Internet (FITNET), was found to be effective in an RCT, leading to significant reduction of fatigue and fatigue-related disabilities [4]. Two-thirds of patients reported fatigue levels and physical functioning within the normal limits, as well as full school attendance following treatment [4]. Currently, in the United Kingdom, an RCT is investigating the feasibility, clinical effectiveness, and cost-effectiveness of FITNET delivered in the context of the National Health Service (FITNET_NHS) [9,10]. FITNET is easily accessible since it is not bound to the geographic location of the therapist delivering the intervention [4]. Patients do not need to travel to a treatment center and can follow the treatment at home, making the intervention easy to follow. Importantly, adolescents with CFS preferred FITNET over face-to-face treatment [4]. It is not self-evident that outcomes of an RCT can be extrapolated to routine clinical care (RCC), since the effectiveness of an RCT may be overestimated due to strict inclusion criteria and close monitoring of the intervention [11,12]. Thus far, the effectiveness of FITNET delivered in RCC has not been shown. Recently, concerns have been raised about the safety of behavioral interventions for CFS/ME. It has been suggested that CBT leads to deterioration of symptoms and functioning [13]. Analyses of RCTs testing the efficacy of CBT for CFS have thus far not shown a higher prevalence of deterioration or more adverse events following or during CBT compared to control conditions [4,14,15]. However, this has not yet been determined for internet-based cognitive behavior therapy (I-CBT) delivered in RCC for adolescents, which uses less strict inclusion criteria and less stringent monitoring of the treatment process.

The primary aim of this study was to determine whether FITNET implemented in RCC (IMP-FITNET) is as effective as in a research context with respect to the outcomes of fatigue severity, physical functioning, school attendance, and recovery rates, using the outcomes of the previous RCT as the benchmark [4]. The secondary aim was to investigate the safety of IMP-FITNET by assessing the frequency of deterioration of fatigue and physical functioning in comparison to a waiting list condition of another benchmark RCT in adolescents [14]. Lastly, the attitude of therapists toward the usability of IMP-FITNET was assessed through semistructured interviews.

## Methods

### Design and Participants

This was an observational study of RCC. Data were collected retrospectively from adolescents who finished treatment in RCC after implementation.

All patients were referred to the Expert Centre for Chronic Fatigue (ECCF), a national referral center for IMP-FITNET and face-to-face CBT for adolescents with CFS/ME, and were retrospectively included in the study between October 2012 and March 2018.

The inclusion criteria were as follows: (1) CFS according to the US Centers for Disease Control (CDC) criteria revised in 2003 [1,16]; (2) 12-18 years of age at baseline; (3) severe fatigue, operationalized as a score of 40 or higher on the fatigue severity subscale of the Checklist Individual Strength-20 (CIS-20) [17]; (4) self-reported substantial disabilities in daily functioning; (5) access to the internet; and (6) no psychiatric comorbidity that could explain the presence of fatigue, ruled out by the Mini International Neuropsychiatric Interview for children (M.I.N.I. KID) [18].

Posttreatment effectiveness in terms of fatigue severity, physical functioning, school attendance, and recovery rates were compared with results derived from the previously published RCT on FITNET [4]. The safety of IMP-FITNET was determined by comparing the prevalence of the deterioration of fatigue and physical functioning with the results of a benchmark study that reported on deterioration rates in a waitlist condition of an RCT testing the efficacy of face-to-face CBT for CFS/ME in adolescents [14]. In RCC, patients received either IMP-FITNET or face-to-face CBT as decided in a shared decision process, in which patients/parents and therapists worked together to choose the best suitable therapy, reflecting both evidence and patient priorities and preferences.

The Dutch Medical Research Involving Human Subjects Act did not apply to our study, as the collected data were part of RCC. Therefore, no formal ethical approval from the medical ethics committee was needed for this study.

### Treatment

CBT for CFS/ME is developed on the basis of a cognitive behavioral model of CFS, assuming that behavior and beliefs can perpetuate symptoms [19]. In this study, two formats of CBT for adolescents with CFS/ME were used. A face-to-face CBT treatment manual for adolescents was applied, which was found to be efficacious in previous research [7]. The second format was an implemented version of the online CBT intervention FITNET with the same content and similar layout, but using a different software package, referred to as IMP-FITNET. FITNET is an internet-based CBT program developed on the basis of the face-to-face CBT treatment manual for adolescents. FITNET has been found to be efficacious in the context of research [4]. The FITNET program consists, aside from psychoeducation on CFS, of 21 interactive treatment modules, with content in line with face-to-face CBT treatment for adolescents and a program for care givers. Each module includes assignments and mandatory e-consultations with the therapist [4,9,20]. IMP-FITNET has the same 21 interactive modules for patients with e-consultations and caregivers. In IMP-FITNET, therapists were however allowed to offer face-to-face consultations or phone calls if deemed necessary, and for patients starting treatment in 2017, videoconferencing was possible.

Eleven trained cognitive behavioral therapists who received weekly supervision from experienced clinical psychologists delivered face-to-face CBT and IMP-FITNET.

### Outcome Variables

#### Primary Outcome Posttreatment

All outcome variables were self-reported. The primary outcome was fatigue severity assessed with the subscale fatigue severity of the CIS-20. This subscale consists of eight items scored on a 7-point Likert scale, resulting in a fatigue severity score ranging from 8 to 56. A score ≥40 indicates the presence of severe fatigue [17]. The internal consistency and discriminative validity of the CIS are excellent [7,17].

#### Secondary Outcomes Posttreatment

Physical functioning was measured with the subscale physical functioning (nine items, range 0%-100%) of the Child Health Questionnaire-87 (CHQ-87). The questionnaire is validated and has good internal consistency [21].

School presence was assessed using a diary and reported as the percentage of classes attended over the past 2 weeks divided by the scheduled number of classes for peers [4].

Recovery was defined in relation to healthy peers by having a CIS-fatigue score <40 [2], a CHQ-87 physical score ≥85% [21], and school absence ≤10% in the past 2 weeks [2]. These recovery criteria were derived from the FITNET RCT [4].

Deterioration of fatigue was defined as an increase of more than six points in CIS-fatigue, and deterioration of physical functioning was defined as a decrease of more than 10 points in CHQ-physical [9,22].

The benchmark for deterioration, as a proxy for safety, was the waiting list condition in a prior RCT on the efficacy of face-to-face CBT [14]. Instead of the CHQ-87 subscale physical functioning, the SF-36 (short-form) of the RAND was used in this study [23].

### Semistructured Interviews

Using a semistructured telephone interview, 11 therapists were asked which criteria they used to propose to start with either IMP-FITNET or face-to-face CBT for the individual adolescent or face-to-face consultations during IMP-FITNET. Nine therapists participated. Interviews with the therapist were recorded and transcribed by one researcher (EA). The themes were independently synthesized by two researchers (EA and LNN) based on the interviews [24]. Discrepancies were resolved through discussion with the principal investigator (HK) to reach consensus. In addition, the number of face-to-face consultations during IMP-FITNET was registered.

### Procedure

After referral, adolescents had two diagnostic face-to-face sessions with a psychologist. The results of the baseline assessment were discussed with the adolescent and parents, and this was followed by a shared decision for either IMP-FITNET or face-to-face CBT. Following treatment, adolescents completed an online posttreatment assessment, which was discussed in a face-to-face session.

### Statistical Analysis

The demographic characteristics of the adolescents and baseline scores were compared using a benchmark strategy, in which the baseline scores of RCC were compared with the 95% CIs of corresponding values in the FITNET RCT. If the mean value in RCC was outside the 95% CI of the RCT, it was considered divergent. The same procedure was used to compare the post-treatment outcomes of RCC with the RCT. Baseline characteristics of patients lost to follow-up were compared with those who were assessed posttreatment using a *t* test for independent groups.

Analyses were based on intention to treat, using the summary estimate of five imputations for 15 missing observations in the primary outcome, with the assumption that data were missing at random [25]. All baseline and posttreatment scores were entered as predictors. The posttreatment score of fatigue severity was entered as a variable to impute. Moreover, analyses were repeated with only those patients who met all the inclusion criteria of the benchmark FITNET RCT (baseline score for physical activity <85% and/or school presence ≤85%). The within-group Cohen *d* was reported as the effect size. For the main outcome fatigue severity, we calculated the percentage of patients who scored below the cutoff of severe fatigue (CIS <40) and reported a reliable change index (z) score greater than +1.96 [26]. To test differences in deterioration, chi-square tests were performed. Percentages of adolescents who were lost to follow-up in the RCT and in RCC were compared with chi-square tests. Lastly, the number of face-to-face consultations during IMP-FITNET were registered.

SPSS version 25 (IBM Corp) was used for statistical analyses, and significance was set at *P*<.05.

## Results

### Study Population

**Figure 1:**
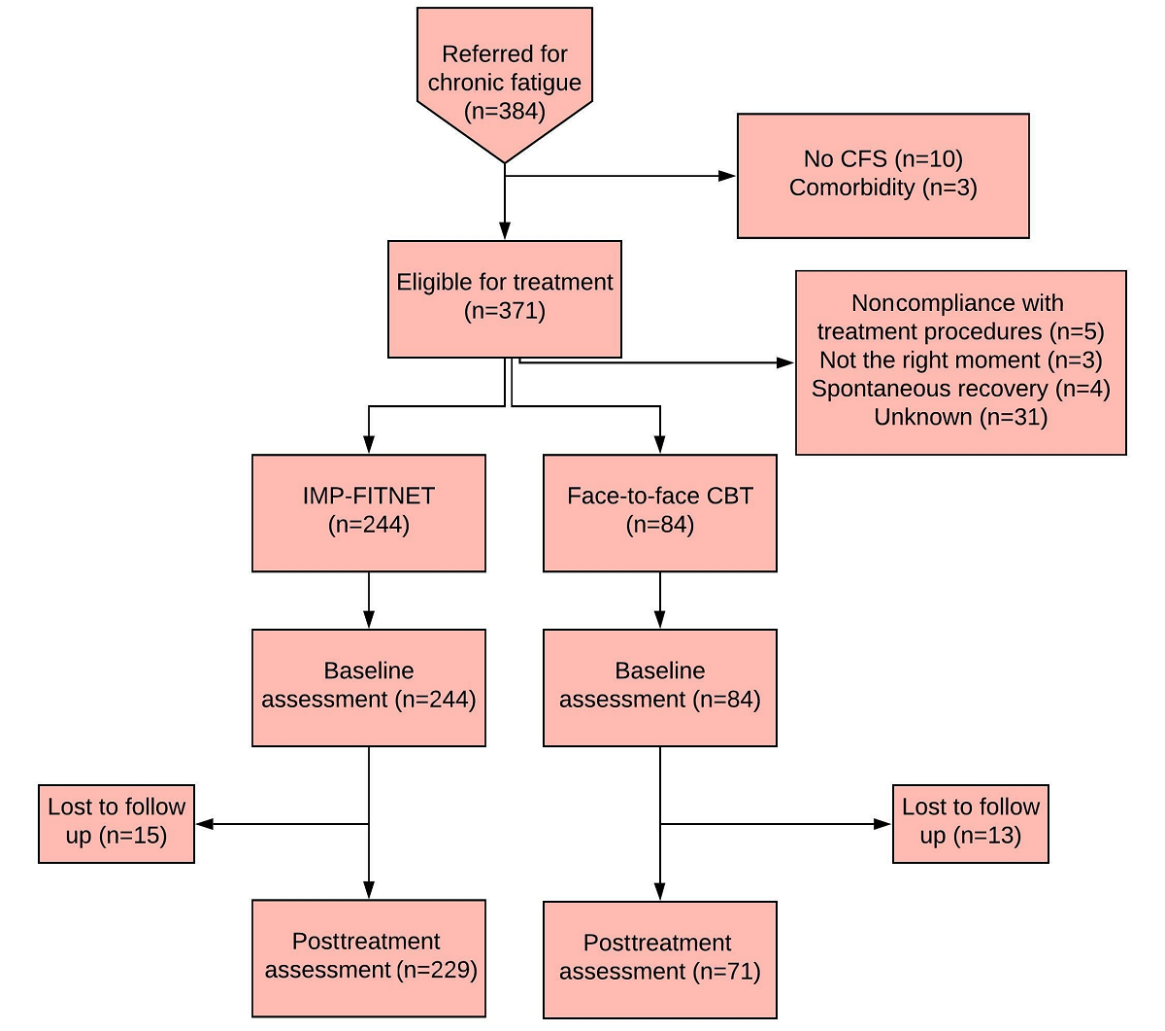
Study flow of the patients in routine clinical care. CBT: cognitive behavioral therapy; CFS: chronic fatigue syndrome; IMP-FITNET: implemented Fatigue in Teenagers on the Internet.

Of the 384 referred adolescents, 371 were eligible for treatment, of which 328 (88.4%) started treatment. Of the 328 patients, 244 (74.4%) received IMP-FITNET and 84 (25.6%) received face-to-face CBT. All 328 adolescents filled out the baseline assessment, 229 of the 244 patients (93.8%) who received IMP-FITNET completed the posttreatment assessment, and 71 of the 84 patients (84.5%) who received face-to-face CBT completed the posttreatment assessment (Figure 1).

### Baseline Characteristics

Adolescents lost to follow-up differed significantly in physical functioning at baseline (mean score 64.7, SD 16.4 vs mean score [lost to follow-up] 73.0, SD 19.4; *t*_241_=−2.04, *P*=.04).

Table 1 provides the baseline characteristics of the adolescents in RCC (IMP-FITNET and face-to-face CBT) compared with the baseline characteristics of the patients from the benchmark FITNET RCT. The duration of symptoms was significantly lower in patients who received IMP-FITNET and significantly higher in patients who received face-to-face CBT compared to the FITNET RCT. Both were outside the 95% CI of the FITNET RCT. The duration of symptoms between IMP-FITNET and face-to-face CBT did not significantly differ (*t*_306_=0.984, *P*=.33). Fatigue severity of the adolescents following either IMP-FITNET or face-to-face CBT was lower and their physical functioning was higher than in the benchmark study, as was their school participation. Moreover, adolescents receiving face-to-face CBT were younger and more often male than in the benchmark study. The analyses were repeated with the subset of patients in RCC who met all the inclusion criteria of the benchmark study. This analysis showed the same pattern of results (data not shown).

The percentage of adolescents lost to follow-up was significantly higher in IMP-FITNET than in the FITNET RCT (8.5% [n=28] vs 3.0% [n=4], N=463, χ^2^_1_=3.3, *P*=.03).

**Table 1:** Baseline characteristics of the patients in routine clinical care and the 95% CIs of the benchmark Fatigue in Teenagers on the Internet randomized controlled trial scores.

Variable	IMP-FITNET^a^ (N=244)	F2F-CBT^b^ (N=84)	95% CI benchmark FITNET RCT^c^ (N=135)^d^
Age at entry (years), mean (SD)	16.1 (1.4)	15.4 (1.8)	15.6-16.1^e^
Gender (female), n (%)	202 (82.8%)	62 (73.8%)	76%-89%^e^
Duration of symptoms at entry (months), median (range)	18 (3-96)	30 (6-96)	20-27 months^e^
Fatigue severity (CIS^f^), mean (SD)	49.8 (5.0)	48.7 (6.3)	50.6-52.2^e^
School attendance, mean % (SD)	60.5 (34.2)	61.1 (33.4)	37.0-47.6^e^
Number of children with >85% school attendance, n/N (%)	70/244 (31.3%)	22/84 (28.9%)	5%-15%^e^
Physical functioning (CHQ-87^g^), mean (SD)	64.7 (16.4)	63.2 (19.3)	55.7-61.8^e^
Anxiety score (STAIC^h^), mean (SD)	31.9 (7.4)	34.7 (8.0)	31.9-34.4^e^

^a^IMP-FITNET: implemented Fatigue in Teenagers on the Internet.

^b^F2F-CBT: face-to-face cognitive behavior therapy.

^c^RCT: randomized controlled trial.

^d^Benchmark FITNET RCT: the study by Nijhof et al [4].

^e^95% CIs of the values of the benchmark FITNET RCT.

^f^CIS: Checklist Individual Strength.

^g^CHQ-87: Child Health Questionnaire-87.

^h^STAIC: State-Trait Anxiety Inventory for Children.

### Primary and Secondary Outcomes Posttreatment

The CIS-fatigue severity score after IMP-FITNET and face-to-face CBT in RCC fell within the 95% CI of the benchmark FITNET study. Additionally, 173 of the 229 adolescents (75.5%) with a posttreatment fatigue score had a reliable change index score greater than +1.96 and a score lower than 40 on the CIS. All secondary outcomes (Table 2) fell within the 95% CIs of the benchmark study. The analyses were repeated with the subset of patients in RCC who fulfilled all the inclusion criteria of the benchmark study. This analysis showed the same pattern of results (data shown in Multimedia Appendix 1).

In RCC, 3 of the 244 patients (1.2%) reported a clinically significant deterioration of fatigue severity after IMP-FITNET. In the waiting list condition of a face-to-face CBT benchmark study, 2 out of 35 patients (5.7%) showed clinically significant deterioration in fatigue severity [15]. This did not significantly differ (N=279; χ^2^_1_=3.5, *P*=.06). Nine of the 244 patients (3.7%) following IMP-FITNET had a baseline score for fatigue severity above 50 and did not show improvement. They could not be identified as patients showing clinically significant deterioration of fatigue due to the ceiling effect of the CIS-20 questionnaire (maximum score of 56).

In RCC, 10 of the 244 patients (4.1%) reported clinically significant deterioration of physical functioning after IMP-FITNET. In the benchmark study [14], 4 of 35 patients (11.4%) showed clinically significant deterioration of physical functioning. Deterioration of physical functioning did not significantly differ between the waiting list condition of the benchmark and IMP-FITNET (N=279; χ^2^_1_=3.3, *P*=.07) (Table 3).

The within-treatment group effect size of FITNET in the RCT was large (Cohen *d*=2.73), with a 95% CI of 2.26 to 3.21. The effect size of IMP-FITNET was also large (Cohen *d*=2.28) and fell within the 95% CI of the FITNET RCT.

**Table 2:** Posttreatment scores of patients in routine clinical care and the 95% CIs of the benchmark Fatigue in Teenagers on the Internet randomized controlled trial scores.

Variable	IMP-FITNET^a^ (N=229)	F2F-CBT^b^ (N=71)	95% CI benchmark FITNET RCT^c^ (N=67)^d^
Fatigue severity (CIS^e^), mean (SD)	26.0 (13.8)	25.8 (12.3)	20.7-27.3^f^
Physical functioning (CHQ-87^g^), mean (SD)	88.2 (15.0)	89.3 (12.8)	85.2-91.9^f^
School attendance, mean % (SD)	84.3 (26.5)	87.1 (23.6)	77.1-91.5^f^
Recovery^h^, %	58%	60%	54-77%^f^

^a^IMP-FITNET: implemented Fatigue in Teenagers on the Internet.

^b^F2F-CBT: face-to-face cognitive behavior therapy.

^c^RCT: randomized controlled trial.

^d^Benchmark FITNET RCT: the study by Nijhof et al [4].

^e^CIS: Checklist Individual Strength.

^f^95% CIs of the values of the benchmark FITNET RCT.

^g^CHQ-87: Child Health Questionnaire-87.

^h^Cutoff scores for recovery are as follows: fatigue severity of <40 on the CIS-20 subscale fatigue; school absence of ≤10%, and a physical functioning score of ≥85% on the CHQ-87 subscale physical functioning.

**Table 3:** Number of patients with symptom deterioration between preassessment and postassessment.

Variable	IMP-FITNET^a^ (N=244)	F2F-CBT^b^ (N=71)	Waiting list condition^c^ (N=35)
Deterioration of fatigue severity^d^, n (%)	3 (1.2%)	2 (2.8%)	2 (5.7%)
Deterioration of physical functioning^e,f^, n (%)	10 (4.1%)	2 (2.8%)	4 (11.4%)

^a^IMP-FITNET: implemented Fatigue in Teenagers on the Internet.

^b^F2F-CBT: face-to-face cognitive behavior therapy.

^c^Data from adolescents on a waiting list condition in a study by Stulemeijer et al [7].

^d^Increase of >6 points on the Checklist Individual Strength (CIS).

^e^Decrease of >10 points on the Child Health Questionnaire-87 (CHQ-87) for patients following IMP-FITNET or F2F-CBT.

^f^Decrease of >10 points on the Short Form-36 (SF-36).

### Semistructured Interviews and Number of Face-to-Face Consultations

Nine of the 11 therapists were interviewed. Face-to-face CBT was preferred to IMP-FITNET when there were interaction problems in the family or when the patient had psychiatric or somatic comorbidities. Therapists decided to make use of face-to-face consultations during IMP-FITNET treatment in the case of perceived inability of the patient to benefit from solely IMP-FITNET or anticipated problems with adherence and motivation. In general, therapists preferred blended therapy with combinations of IMP-FITNET.

Of the 244 adolescents who started IMP-FITNET, 116 (47.5%) followed only IMP-FITNET without face-to-face consultations and 102 (41.8%) had at least one face-to-face consultation with their therapist, and of these, 41 adolescents (16.8%) had over 3 face-to-face consultations. Adolescents who used face-to-face consultations had on average about three face-to-face consultations (mean 3.2, SD 3.81, modus 1). Moreover, videoconferencing became an additional feature during IMP-FITNET treatment for 50 patients, of which 23 patients (46.0%) used videoconferencing, with an average of 5.1 conferences (SD 3.48) lasting on average 24.3 minutes (SD 20.7).

## Discussion

The posttreatment outcomes of adolescents with CFS/ME treated with I-CBT implemented in RCC (IMP-FITNET) were within the CIs of the outcomes from the benchmark with respect to levels of fatigue severity, physical functioning, school attendance, and recovery rates at posttreatment. Additionally, 133 of the 229 (58.1%) adolescents treated with IMP-FITNET met the recovery criteria posttreatment. The within-treatment group effect size of the decrease in fatigue severity with IMP-FITNET was also within the CI of the benchmark. At baseline, patients had an average fatigue severity score of 49.8 (SD 5.0), and after treatment, their fatigue severity score reduced on average by 23.8 points to 26.0 (SD 13.8). We conclude from this that IMP-FITNET applied in RCC is an effective intervention. Our findings are in line with the results of studies in adult patients with CFS/ME, in which blended CBT implemented in RCC was as effective as in a research context [27,28].

The primary and secondary outcomes of adolescents following face-to-face CBT were also within the CIs of the FITNET RCT. A quarter of the referred patients eligible for IMP-FITNET started with face-to-face CBT after a shared decision process. This was more often the case when patients were young, were male, had a long symptom duration, and were anxious. Therapists indicated preferring face-to-face CBT in case of family interaction problems, psychiatric or somatic comorbidities, and problems with motivation.

The prevalence of a clinically significant deterioration following IMP-FITNET was low in general and comparable with a waiting list condition of a prior CBT study [14]. Therefore, we consider IMP-FITNET safe for application in RCC, without evidence of an increased risk of deterioration in fatigue and physical functioning. This replicates similar findings in previous studies of the safety of CBT for CFS/ME conducted in a research context [14].

At baseline, differences existed between adolescents who received IMP-FITNET and those in the benchmark FITNET RCT. Adolescents in RCC were less severely fatigued, were less physically impaired, had less school absence, and had a shorter symptom duration compared with patients from the benchmark RCT. This may be the result of the increased availability of an evidence-based and internet-based treatment for this patient group after nationwide implementation of I-CBT that followed the publication of the FITNET RCT results. Moreover, for these less severely affected adolescents with CFS/ME, IMP-FITNET is effective. IMP-FITNET has the advantage that adolescents do not need to travel for treatment. The nationwide availability of an effective intervention favors earlier referral.

This clinical observational study was not designed to investigate the difference in effectiveness of IMP-FITNET versus face-to-face CBT in relation to specific patient populations. Nevertheless, we found that even with a less strict treatment protocol and a more blended form of treatment, IMP-FITNET is effective. Although CBT for adolescents with CFS/ME (FITNET, face-to-face CBT, or IMP-FITNET) is considered effective, one-third of patients do not recover. To further improve the treatment and prognosis of adolescent CFS/ME, it is important to identify the factors that contribute to treatment effectiveness and assess which factors are associated with nonrecovery. Some issues need further consideration. First, in this observational study design, the choice of the treatment form (face to face vs IMP-FITNET) was determined by health care providers taking into account the patient’s preference. For this reason, there are methodological limitations, and the most important one is the risk of confounding by indication [29]. Second, the inclusion criteria of the FITNET RCT were stricter than those of IMP-FITNET, applying cutoff scores for physical functioning or school participation [4]. We did not find evidence that this influenced the outcomes of IMP-FITNET as the pattern of results was similar in the subgroup of patients who followed IMP-FITNET and met the stricter inclusion criteria. Next, the average time between pretreatment and posttreatment assessments in RCC was much longer than in the FITNET RCT, owing to the waiting list, holidays, and breaks. Third, not all results of the RCT could be compared with the findings of this study since some data were obtained with a different method or were not systematically assessed in the implementation study (eg, self-reported recovery). Fourth, although still relatively low, the dropout rate in RCC was significantly higher than in the FITNET RCT. This could, despite imputation of missing data, have led to selection bias. Lastly, the benchmark used to compare deterioration of physical functioning did not use the exact same questionnaire. As this study reported on data from patients who were treated with an evidence-based treatment in RCC, we do attribute the reduction in symptoms to the treatment with IMP-FITNET. Moreover, the advantage of retrospective data from RCC is the unbiased representation of the patient population.

IMP-FITNET in RCC was adapted according to therapist and patient preferences for video or face-to-face consultations. A substantial number of adolescents who followed IMP-FITNET had one or more face-to-face consultations. A blended form of IMP-FITNET, in which different modalities of communication can be used, may have advantages and is in line with the current practice to combine internet interventions with face-to-face interaction with a therapist. One limitation is that during implementation of the FITNET treatment, technical options were expanded, for example, video consultations were integrated in the portal. The increasingly rapid development within software systems makes it more difficult to compare treatments designed at different time points. More research is necessary to inform when blended CBT is more effective than internet-based treatment alone. Further research also has to show whether blended care, with video consultations, is as cost-effective as FITNET with only email contact.

In conclusion, this study showed that IMP-FITNET is an effective and safe treatment for adolescents with CFS/ME in RCC. In RCC, the therapist can tailor the mode of delivery of the intervention to the needs of the individual patient.

## Appendix

Multimedia Appendix 1 Posttreatment scores of the 226 patients in routine clinical care who fulfilled all the inclusion criteria of the Fatigue in Teenagers on the Internet randomized controlled trial.

## Abbreviations

CBT: cognitive behavior therapy
CFS: chronic fatigue syndrome
CHQ-87: Child Health Questionnaire-87
CIS-20: Checklist Individual Strength-20
FITNET: Fatigue in Teenagers on the Internet
I-CBT: internet-based cognitive behavior therapy
IMP-FITNET: implemented Fatigue in Teenagers on the Internet
ME: myalgic encephalomyelitis
RCC: routine clinical care
RCT: randomized controlled trial
