# Supplementary material for: Effectiveness of Internet-Based Cognitive Behavior Therapy (Fatigue in Teenagers on the Internet) for Adolescents With Chronic Fatigue Syndrome in Routine Clinical Care: Observational Study
Source: J Med Internet Res. 2021 Aug 13;23(8):e24839. doi: 10.2196/24839 (PMC8398746; doi:10.2196/24839)
Supplement: Multimedia Appendix 1 [file jmir_v23i8e24839_app1.docx]

**Multimedia Appendix 1.** Posttreatment scores of the 226 patients in routine clinical care who fulfilled all the inclusion criteria of the Fatigue in Teenagers on the Internet randomized controlled trial.

|  | IMP-FITNET^a^ (N=226)^e^ |
| --- | --- |
| Fatigue severity^b^, mean (SD) | 25.7 (13.9) |
| Physical functioning^c^, mean (SD) | 88.2 (14.8) |
| School attendance, mean % (SD) | 83.5 (27.0) |
| Recovery^d^, % | 58% |

^a^ IMP-FITNET: implemented – Fatigue in Teenagers on the interNET

^b^ CIS: Checklist individual strength

^c^ CHQ-CF87: Child health questionnaire

^d^ Cut-off scores for recovery are: fatigue severity of less than 40 on the CIS-20 subscale fatigue; school absence of 10% or less and a physical functioning score of 85% or more on the CHQ-CF87 subscale physical functioning.

^e^ 226 patients did fulfil the inclusion criteria of the FITNET RCT at baseline, 18 patients did not fulfil the inclusion criteria of the FITNET RCT at baseline.
